# Pneumonie varicelleuse du nouveau-né: à propos ďun cas

**Published:** 2012-10-03

**Authors:** Jean Jacques Nzeale Noubiap, Ulrich Gaël Tene, Adamo Bongoe

**Affiliations:** 1 Service de Médecine Interne, Hôpital Régional Annexe ďEdéa, Edéa, Cameroun; 2 Service de Pédiatrie, Hôpital Régional Annexe ďEdéa, Edéa, Cameroun; 3 Service Gynécologie-Obstétrique, Hôpital Régional Annexe ďEdéa, Edéa, Cameroun

**Keywords:** Varicelle, grossesse, nouveau-né, pneumonie, aciclovir, varicella, pregnancy, newborn, pneumonia, acyclovir

## Abstract

La varicelle est une maladie contagieuse fréquente chez ľenfant, mais rare chez la femme enceinte. La survenue de varicelle pendant la grossesse peut entrainer des complications périnatales dont la pneumonie varicelleuse du nouveau-né. Cette atteinte pulmonaire est accompagnée ďun taux élevé de décès. Nous rapportons un cas de pneumonie varicelleuse grave chez un nouveau-né qui a été contaminé par le virus de la varicelle par voie transplacentaire. Le tableau clinique associait un syndrome infectieux, une détresse respiratoire sévère avec coma, des râles sous-crépitants diffus aux deux champs pulmonaires, et une éruption cutanée disséminée faite de macules, vésicules, croûtes, évocatrice de la varicelle. La radiographie du thorax montrait un syndrome interstitiel diffus aux deux poumons. Un traitement par ľaciclovir injectable associé à ľoxygénothérapie continue a permis une évolution vers la guérison. La pneumonie varicelleuse du nouveau-né est situation associée à une forte mortalité mais dont le traitement par ľaciclovir injectable peut permettre la guérison. La prophylaxie par administration intraveineuse ďaciclovir ou ďimmunoglobulines polyvalentes chez le nouveau-né permet de diminuer la sévérité et la mortalité de la varicelle périnatale.

## Introduction

L'infection par le virus de la varicelle, le virus varicelle-zona (VZV), atteint préférentiellement les enfants, mais peut également atteindre les adultes [1]. Le risque de survenue d'une varicelle pendant la grossesse est assez faible, de l'ordre de 1 à 5 cas pour 10.000 grossesses [2]. La varicelle pendant la grossesse peut entraîner des complications chez la mère, chez le fœtus (fœtopathie varicelleuse), et le nouveau-né (varicelle périnatale). La varicelle périnatale est associée à un taux élevé de décès lorsque la maladie se déclenche chez la mère dans les 5 jours qui précèdent et les 48 heures qui suivent l'accouchement [3, 4]. L'atteinte pulmonaire constitue dans ces cas la principale cause de décès, avec un taux de mortalité qui avoisine les 30% [3, 4]. Nous rapportons un cas de pneumonie varicelleuse grave chez un nouveau-né.

## Patient et observation

Les auteurs déclarent avoir reçu le consentement des parents du patient pour la publication de ce cas. Il s'agit d'un nouveau-né de sexe masculin, que nous avons reçu à son 10^ème^ jour de vie pour difficultés respiratoires. Il est né à la suite d'une grossesse bien suivie et sans particularités jusqu’à la 38^ème^ semaine. A la 38^ème^ semaine de grossesse et 4 jours avant l'accouchement, sa mère, séronégative au VIH et n'ayant jamais eu la varicelle auparavant, a présenté un exanthème prurigineux fait de lésions initialement maculopapuleuses, puis vésiculeuses, pustuleuses et croûteuses; ces lésions évoquaient une varicelle. L'accouchement s'est fait normalement par voie basse, et l'enfant avait un bon état à la naissance. Il a été normalement mis au sein. Aucune prophylaxie contre la transmission de la varicelle de la mère à l'enfant n'a été réalisée.

**Figure 1:**
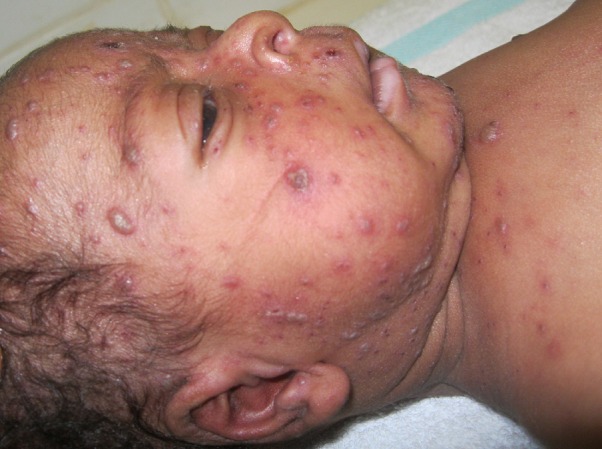
Exanthème typique de la varicelle fait de macules, papules, vésicules et croûtes

A partir de son septième jour de vie, il a présenté une éruption cutanée associée à une fièvre non chiffrée. Au troisième jour de cette éruption cutanée, il s'est installé une détresse respiratoire d'aggravation rapide motivant une consultation médicale aux urgences de l'Hôpital Régional Annexe d'Edéa. A son arrivée, le nouveau-né était inconscient, sa température après administration de paracétamol à la maison était de 37°C. Il présentait une détresse respiratoire sévère, avec un score de Silverman de 9/10, et une fréquence respiratoire de 64cyles/minute. L'auscultation pulmonaire révélait des râles sous-crépitants diffus aux deux champs pulmonaires. Hormis une tachycardie à 132cycles/minute, l'examen cardiaque était normal; le temps de recoloration cutané était d'environ 2secondes. La peau présentait des macules, des papules, des vésicules sur fond érythémateux, ainsi que des croûtes, lésions caractéristiques de la varicelle (Figure 1). Les muqueuses et les téguments étaient bien rosées; il n'y avait pas d'ictère.

**Figure 2:**
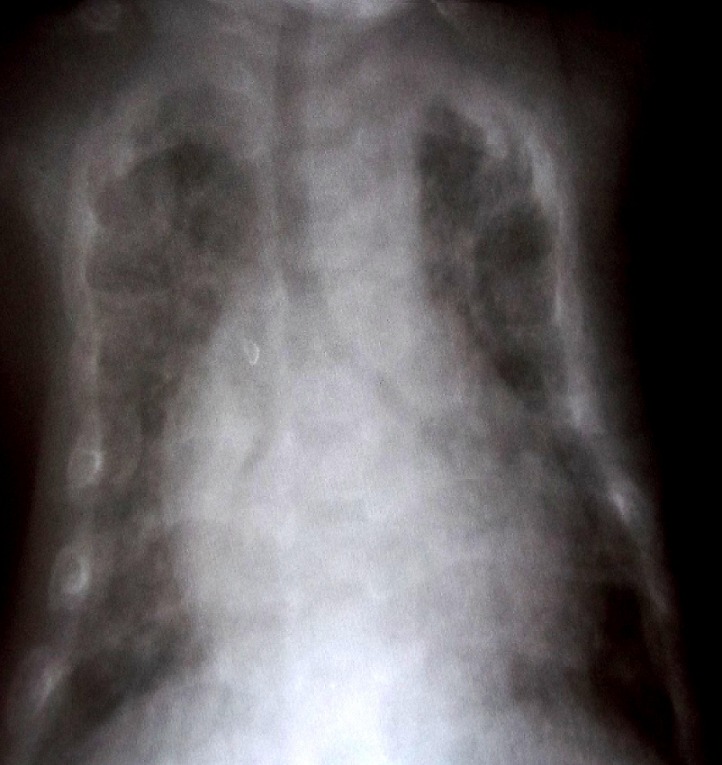
Radiographie du Thorax: Syndrome interstitiel diffus aux deux poumons

**Figure 3:**
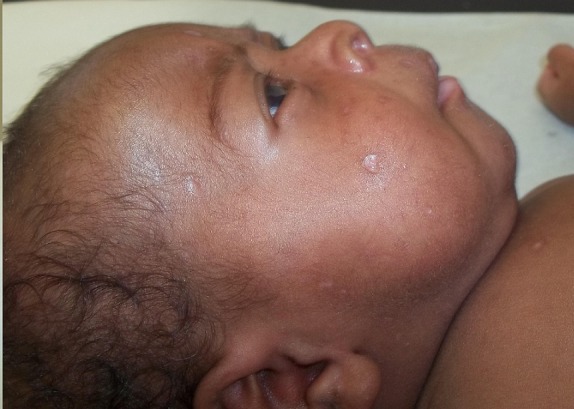
Peau après guérison de la varicelle

A la biologie, la CRP (C Reactive Protein) était élevée à 56mg/l; la numération formule sanguine était normale. La radiographie du thorax montrait un syndrome interstitiel diffus aux deux poumons (Figure 2). Ces éléments cliniques et radiologiques nous ont conduits à poser le diagnostic de pneumonie varicelleuse compliquée de détresse respiratoire sévère et de coma. En guise de traitement, il a été mis sous oxygénothérapie à 2l/min, aciclovir injectable 20mg/kg/8h, ceftriaxone 50mg/kg/24h et paracétamol injectable 15mg/kg/6h. A cause des moyens financiers limités des parents, l'aciclovir a été administrée pendant seulement 3 jours. L’évolution a été marquée par une diminution significative de la détresse respiratoire et des râles sous-crépitants à partir du 3^ème^ jour de traitement. Au 4^ème^ jour, il pouvait à nouveau téter. A sa sortie, au 5^ème^ jour d'hospitalisation, l’état général était quasiment normal; les lésions cutanées étaient en voie de cicatrisation pour la plupart (Figure 3).

## Discussion

La survenue d'une varicelle pendant la grossesse est assez rare, avec une prévalence de 1 à 5 pour 10.000 grossesses [2]. La varicelle pendant la grossesse peut entraîner des complications chez la mère et chez le fœtus (fœtopathie varicelleuse) ou le nouveau-né (varicelle périnatale). En cas de varicelle périnatale, il peut s'agir soit d'une forme congénitale, lorsque l′infection a été contractée in utero, soit une varicelle acquise après l′accouchement. La varicelle congénitale survient au cours des 10 premiers jours de vie. Au-delà du 10^ème^ jour, il s'agit d'une varicelle acquise après l'accouchement [5]. Ainsi, dans notre cas, la survenue du rash cutané chez la mère 4 jours avant l'accouchement et au 7^ème^ jour de vie chez le nouveau-né prouve qu'il s'agit d'une forme congénitale, c'est-à-dire résultant d'une transmission materno-fœtale par voie transplacentaire.

La varicelle chez le nouveau-né est généralement sévère, avec un taux de mortalité qui avoisine 30%; l'atteinte pulmonaire est la principale cause de décès [3–5]. La sévérité de la varicelle congénitale et la mortalité associée sont corrélables à la date de survenue du rash cutané. Le risque de décès par pneumonie varicelleuse est nul si l′éruption survient chez la mère 5 jours ou plus avant l′accouchement, ou bien si la varicelle néonatale survient avant le 4^ème^ jour de vie. Il est de 30% quand la varicelle apparaît chez la femme enceinte 4 jours avant l′accouchement, il est de 20% quand le rash apparaît, chez le nouveau-né, à 5 jours de vie ou plus [5]. Ainsi, compte tenu des dates de survenue du rash cutané chez notre nouveau-né et sa mère le risque mortalité par pneumonie varicelleuse chez lui était d'au moins 20 à 30%. En effet, lorsque la varicelle survient quelques jours avant l'accouchement, les anticorps protecteurs maternels n'ont pas eu le temps d’être produits, ou bien d’être transmis au fœtus via le placenta. N'ayant pas d'anticorps protecteurs et son système immunitaire étant immature, le nouveau-né se trouve ainsi particulièrement à risque.

Le traitement curatif étiologique de la pneumonie varicelleuse chez le nouveau-né fait appel à l'aciclovir injectable à raison de 20mg/kg/6h pendant 5 jours [6]. Une des difficultés dans la prise en charge de notre nouveau-né a été la non disponibilité de l'aciclovir injectable dans notre hôpital et dans toute la ville d'Edéa. Ce médicament n'a pu être trouvé que dans l'une des pharmacies de Douala, l'une des deux grandes villes du Cameroun. A cause de son coût élevé et des moyens financiers limités des parents, l'enfant n'a reçu de l'aciclovir injectable que pendant 3 jours. En absence de forme injectable, la forme orale ne peut apporter qu'un bénéfice nettement moindre. Car à cause de sa faible biodisponibilité par voie orale (seulement 20% du produit sont résorbés), la concentration d'aciclovir nécessaire pour inhiber la réplication virale in vitro est difficilement obtenue par voie orale [6].

Malgré un risque minimal de décès théoriquement estimé à 30% chez notre nouveau-né, en plus de la sévérité de l'atteinte pulmonaire comme le témoignaient la détresse respiratoire sévère et l'altération de la conscience à l'entrée, l’évolution sous traitement s'est faite vers la guérison. Les trois jours de traitement à l'aciclovir injectable, associé à une antibiothérapie à large spectre pour prévenir une éventuelle surinfection bactérienne, ainsi que l'oxygénothérapie en continue, ont permis cette évolution favorable.

En conformité avec les recommandations en la matière, une prophylaxie aurait pu être administrée à notre nouveau-né dès sa naissance. Cette prophylaxie s'applique aux nouveau-nés de mères qui ont développé une varicelle dans les 5 jours qui ont précédé et les 48 heures qui ont suivi l'accouchement [6]. Cette prophylaxie fait appel à l'aciclovir par voie intraveineuse à la posologie de 500mg/m^2^ de surface corporelle/8h sur une durée moyenne de 10 jours, ou aux immunoglobulines polyvalentes par voie intraveineuse à la posologie de 4 mL/kg/24 h en une prise [6]. Cette prophylaxie permettrait de diminuer la sévérité et la mortalité de la varicelle périnatale.

## Conclusion

La varicelle périnatale est une affection peu fréquente, mais dont la mortalité est très élevée. La pneumonie varicelleuse en est la principale complication et la principale pourvoyeuse de décès. L'aciclovir injectable est le médicament de base à visée étiologique. La prophylaxie par administration intraveineuse d'aciclovir ou d'immunoglobulines polyvalentes chez le nouveau-né permet de diminuer la sévérité et la mortalité de la varicelle périnatale.

## References

[1] Baren JM, Henneman PL, Lewis RJ (1996). Primary varicella in adults: Pneumonia, Pregnancy, and Hospital Admission. Ann Emerg Med.

[2] Siegel M, Fuerst HT (1966). Low birth weight and maternal virus diseases: a prospective study of rubella, measles, mumps, chickenpox and hepatitis. JAMA.

[3] Brunell PA (1983). Fetal and neonatal varicella zoster infections. Semin Perinatol.

[4] Preblud SR, Bregman DJ, Vernon LL (1985). Deaths from varicella in infants. Pediatr Infect Dis.

[5] Meyers JD (1974). Congenital varicella in term infants: risk reconsidered. J Infect Dis.

[6] Ovetchkine P (2007). Varicelle. Encycl Med Chir (Maladies infectieuses).

